# Evaluation of an Online Platform for Multiple Sclerosis Research: Patient Description, Validation of Severity Scale, and Exploration of BMI Effects on Disease Course

**DOI:** 10.1371/journal.pone.0059707

**Published:** 2013-03-20

**Authors:** Riley Bove, Elizabeth Secor, Brian C. Healy, Alexander Musallam, Timothy Vaughan, Bonnie I. Glanz, Emily Greeke, Howard L. Weiner, Tanuja Chitnis, Paul Wicks, Philip L. De Jager

**Affiliations:** 1 Program in Translational NeuroPsychiatric Genomics, Department of Neurology, Institute for the Neurosciences, Brigham and Women’s Hospital, Boston, Massachusetts, United States of America; 2 Department of Neurology, Partners MS Center, Center for Neurologic Diseases, Brigham and Women’s Hospital, Boston, Massachusetts, United States of America; 3 Harvard Medical School, Boston, Massachusetts, United States of America; 4 PatientsLikeMe, Inc., Cambridge, Massachusetts, United States of America; 5 Program in Medical and Population Genetics, Broad Institute, Cambridge, Massachusetts, United States of America; University Hospital La Paz, Spain

## Abstract

**Objectives:**

To assess the potential of an online platform, PatientsLikeMe.com (PLM), for research in multiple sclerosis (MS). An investigation of the role of body mass index (BMI) on MS disease course was conducted to illustrate the utility of the platform.

**Methods:**

First, we compared the demographic characteristics of subjects from PLM and from a regional MS center. Second, we validated PLM’s patient-reported outcome measure (MS Rating Scale, MSRS) against standard physician-rated tools. Finally, we analyzed the relation of BMI to the MSRS measure.

**Results:**

Compared with 4,039 MS Center patients, the 10,255 PLM members were younger, more educated, and less often male and white. Disease course was more often relapsing remitting, with younger symptom onset and shorter disease duration. Differences were significant because of large sample sizes but small in absolute terms. MSRS scores for 121 MS Center patients revealed acceptable agreement between patient-derived and physician-derived composite scores (weighted kappa = 0.46). The Walking domain showed the highest weighted kappa (0.73) and correlation (rs = 0.86) between patient and physician scores. Additionally, there were good correlations between the patient-reported MSRS composite and walking scores and physician-derived measures: Expanded Disability Status Scale (composite rs = 0.61, walking rs = 0.74), Timed 25 Foot Walk (composite rs = 0.70, walking rs = 0.69), and Ambulation Index (composite rs = 0.81, walking rs = 0.84). Finally, using PLM data, we found a modest correlation between BMI and cross-sectional MSRS (rho = 0.17) and no association between BMI and disease course.

**Conclusions:**

The PLM population is comparable to a clinic population, and its patient-reported MSRS is correlated with existing clinical instruments. Thus, this online platform may provide a venue for MS investigations with unique strengths (frequent data collection, large sample sizes). To illustrate its applicability, we assessed the role of BMI in MS disease course but did not find a clinically meaningful role for BMI in this setting.

## Introduction

Investigations of factors associated with progressive disease in multiple sclerosis (MS) have been hampered by the variable and slow course of the disease, a lack of robust biomarkers and the costs of generating large datasets longitudinally. The emergence of online patient-centered research platforms holds great promise for certain forms of clinical research by providing rapid access to data from large numbers of patients at frequent intervals. However, the quality and validity of data collected online must be assessed critically.

PatientsLikeMe (www.patientslikeme.com; PLM) is an online platform that includes 29,570 subscribers reporting having MS (as of October 2012). In addition to personal and disease characteristics, individuals also report their disease severity using an MS Rating Scale (MSRS) designed by PLM [1]. Currently, while several validated patient-reported outcome measures of MS disease severity exist, some with online platform experience as in the case of the North American Research Committee on Multiple Sclerosis (NARCOMS) [2]–[4], there is no “gold standard” for data capture through online platforms. The MSRS has previously undergone psychometric validation and shown high internal consistency, concordant validity with the NARCOMS Patient-Derived Disease Step, and demonstrated adequate known-groups validity. The instrument has been made available for free by PatientsLikeMe under Creative Commons license [1].

To determine the potential and limitations of this platform for future investigations, our study aimed first to determine whether PLM members reporting MS were comparable to patients with MS followed at a large regional MS referral center. Then, the MSRS was validated in a clinical setting. Finally, the potential of this platform was tested by assessing the role of body mass index (BMI), a risk factor for MS [5]–[7], on disease course, a phase of the disease for which limited data exist in this context [8].

## Methods

### I. Comparison of Demographic and Disease Characteristics of PLM Members with Patients from the Partners Multiple Sclerosis Center

#### Subjects

PLM is an online research platform based in Cambridge, Massachusetts, designed to allow patients to share data about their conditions, treatments, symptoms and comorbidities through structured data collection [9], but with some features of an online social network. Members join the site with the explicit understanding that any data provided will be shared anonymously but openly, including with pharmaceutical manufacturers, from which the company derives its funding. Use of the system has been associated with positive measures of patient literacy, communication and support [10], with development of other patient-reported outcomes [11]–[13], and with clinical insights [14]. From the over 29,570 members of PLM reporting a diagnosis of MS, members aged 18 and above were selected if (1) they provided at least 4 of the 5 following baseline characteristics: age, sex, disease type, age at first symptom, age at diagnosis; and (2) they had updated their profiles at least twice between 1/1/2009 and 8/31/2011 (n = 10,255). Both demographic (age, race, ethnicity, gender, family history of MS, education) and disease characteristics (disease type, age at first symptoms, use of disease modifying therapy (DMT)) were obtained from the PLM databases.

The Partners MS Center (Brigham and Women’s Hospital) is a regional MS referral center in the Northeastern United States. Detailed electronic records of patient visits to the Center are captured using structured encounter forms and stored within an Oracle software-based relational database designed to follow the longitudinal histories of patients with demyelinating diseases. Data derived from this cohort have been extensively described [15] **.** We included all subjects aged 18 or older, with an MS diagnosis by 2005 McDonald Criteria, who were evaluated clinically at least twice at the Partners MS Center since 1/1/2009 (n = 4,039). IRB approval was obtained for the analysis of the existing PLM and Partners data from the Partners Healthcare Human Research Committee, our Institutional Review Board. Given the consent documents governing the use of the Partners MS center data, we could not determine whether any MS Center patients were also members of PLM.

#### Statistical analysis

T-tests were used for continuous outcomes and chi-squared analyses were used for categorical outcomes. Identical analyses were performed for subsets of patients treated with glatiramer acetate and interferons. Due to missing data for certain individuals, percentages for each variable were calculated based on a different sample sizes.

### II. Validation of the Patient-reported MSRS

#### Subjects

Patient- and physician-derived MSRS scores were compared for a sample of MS patients seen at the Partners MS Center over 20 days in November 2011. All patients aged 18 or older, who carried a diagnosis of MS based on 2005 McDonald Criteria by an MS Center physician, were fluent in English, and who presented for routine neurological follow up at the Partners MS Center were approached. IRB approval was obtained from the Partners Healthcare Human Research Committee. Verbal informed consent was obtained from participants, in compliance with the IRB, because it was determined that the research presented no more than minimal risk of harm to subjects and involved no procedures for which written consent is normally required outside the research context. A study fact sheet describing the study was provided with the questionnaire to each patient approached, in compliance with the IRB. Thus only consenting individuals would complete the questionnaire, and blank questionnaires would be stored for those who did not consent. None of the 162 patients who were approached refused participation. Of these patients, 34 were unable to participate due to the constraints of the MS Center schedule for clinical encounters. In addition, 7 patients who filled out the questionnaire did not have a definite MS diagnosis. This resulted in 121 subjects and their physician completing an MSRS evaluation.

#### MSRS score

In this 7-item questionnaire, patients rate their disability on a 0–4 scale in 7 areas (walking, use of upper extremities, speech disturbance, vision, dysphagia, cognitive or affective disturbance, and sensory disturbance). Total scores range from 0–28 (**Table S1**).

#### Clinical assessment

Patients completed the MSRS independently of the physician prior to the clinical encounter. Following the clinical encounter, each patient’s physician then completed the MSRS form, which covers functions routinely assessed and logged in the Oracle database, and then provided scores for the Expanded Disability Status Scale (EDSS) [16], Ambulation Index (AI [17]) and Timed 25 foot walk (T25FW [18]).

#### Statistical analysis

For comparisons of patient- and physician-derived MSRS scores, a weighted kappa statistic was calculated for each MSRS domain, as well as for the composite (sum) score to assess the agreement between the two raters. For the composite score, a Bland-Altman plot for the patient and physician score was also created. In addition, Spearman’s correlation coefficient was also calculated to assess the level of association between the two raters. Finally, the proportion of agreement between physician and patient was calculated for each MSRS domain, and reason for lack of concordance (physician rates score higher, vs. patient rates score higher) was recorded. Nonparametric Spearman correlation coefficients were also calculated between EDSS, T25FW, and AI and both patient- and physician-derived MSRS composite and walking domain subscores. This allowed us to assess the criterion validity (the extent to which the measure of interest corresponds with an existing external criterion [19]) of the MSRS walking domain subscore.

### III. Investigation of BMI on MS Disease Course in PLM

#### Subjects

PLM members were selected in an identical manner to (I), but with profiles updated at least twice between 1/1/2009 and 1/1/2012 (n = 10,433). IRB approval was obtained for analysis of these existing PLM data from the Partners Healthcare Human Research Committee.

#### Body mass index

Self-reported height, weight, and calculated BMI (kg/m2) were included for all PLM subjects in the study. The following measurements were excluded: height <55 or >85 inches, weight <70 or >540 lbs, and BMI >190.

#### Statistical analysis

BMI was first classified according to WHO criteria to examine the distribution of this parameter in our sample: underweight (<18.50 kg/m^2^), normal weight (18.50–24.99 kg/m^2^), overweight (25.00–29.99 kg/m^2^), and obese (≥30.00 kg/m^2^). Then, a cross-sectional analysis was performed correlating continuous BMI at most recent data entry point with MSRS composite score, using nonparametric correlations and adjusting for age, sex, race, disease duration, and disease type. Third, to assess the impact of BMI on longitudinal accumulation of disability (MSRS), we used two mixed models with random slopes and intercepts and robust standard errors to estimate the interaction between BMI and follow-up time, controlling for age, sex, race, disease duration, and disease type, and the interaction between disease type and follow-up time. In the first model, the predictor of interest was the earliest BMI available for each subject after symptom onset. In the second model, the predictor of interest was BMI within the first three months of symptom onset, which was available for a subset of subjects.

Only de-identified PLM data were transferred to the Brigham and Women’s Hospital research team for quality control and analysis. All statistical analyses were performed using either the R version 2.14.2 (www.r-project.org) or SAS Software (Version 9.3).

## Results

### I. Comparison of Subject and Disease Characteristics between PLM Members and Partners MS Center Subjects

In comparing how an online patient population recruited at a national level (PLM members) relates to a patient population found at a large regional MS center (the Boston-based Partners MS Center patient population), we found small (in absolute terms) but statistically significant differences for all tested variables (p<0.0001 for all comparisons, **Table 1**). Specifically, PLM members were younger, more educated, more often female, and less often white. Further, the reported disease course of PLM members was more often relapsing remitting, with younger age at symptom onset and shorter disease duration. A family history of MS was also less common among PLM members. Similar proportions of PLM and MS Center subjects used glatiramer acetate (24% vs. 25%), but more PLM subjects used interferon beta (29% vs. 18%). For both treatment groups, the differences in demographic characteristics between the MS Center and PLM populations were similar to those reported between the MS Center and the complete PLM population examined (**Table S2**).

**Table 1:** Comparison of PLM members' individual and disease characteristics with those of patients followed at the Partners MS Center.

Variable	PLM	MS Center	Comparison
	N = 10255	N = 4039	p-value
	Mean (SD)	Mean (SD)	
Current Age, yrs	44.8 (10.6)	47.8 (12.0)	<0.0001
Age at first symptom, yrs	32.8 (10.0)	34.2 (10.8)	<0.0001
Disease Duration, yrs	12.0 (9.3)	13.7 (10.5)	<0.0001
	**%**	**%**	
Gender (% F)	80.1	74.8	<0.0001
Family History of MS (% yes)	22.4	25.5	0.0002
**Race**			<0.0001
White	90.4	92.4	
African American	5.1	4.6	
Other	4.5	3.0	
**MS course**			<0.0001
Relapsing-remitting	77.6	70.2	
Secondary progressive	12.0	22.6	
Primary progressive	7.3	6.2	
Progressive relapsing	3.1	1.0	
**Education Level**			<0.0001
Less than 12 years	2.2	2.5	
Completed High School	14.7	16.9	
Some College	42.2	54.8	
Completed College	25.8	12.2	
Post Graduate	15.1	13.6	

NB. For individual variables, as some subjects had missing data, the N used for calculation of percentages was lower than the total N of respondents.

### II. Validation of Patient Reported MSRS

To assess the validity of the patient reported MSRS instrument deployed in PLM’s online platform, we administered this instrument to 121 MS patients at the Partners MS Center, with comparable demographic and disease characteristics to the entire MS Center population described in **Table 1** (**Table S3)**, and with the spectrum of disability seen at our Center (median EDSS: 2, range: 0–8.5).

Good agreement between the patient- and physician-derived composite MSRS scores was observed (weighted kappa = 0.43, **Table 2** **; Figure S1**). The two scores were strongly correlated (Spearman rank-order correlation coefficient, r_s_ = 0.693). When we examined individual domains, the walking score had the highest level of agreement (weighted kappa = 0.732) and the highest level of correlation (r_s_ = 0.856). There was also good agreement and association for upper extremity weakness and swallowing (weighted kappa >0.4, r_s_>0.5). The other four domains had lower levels of agreement between physician and patient assessments but all were significantly correlated (p<0.0001). The relationships between the patient and physician scores for each domain are shown in **Table 2** and **Table S4a–g**. There did not appear to be any systematic under- or over-reporting of symptoms by either patients or physicians, as evidenced by the percentages of patients vs. physicians providing a higher score (**Table 2**).

**Table 2:** Comparison of patient- and physician- scored MSRS.

	AGREEMENT BETWEEN PATIENT AND PHYSICIAN (% Scores)			Weighted kappa statistic	Correlation	
Domain	Patient rated higher	Perfect Agreement	Patient rated lower	(95% CI)	Spearman r	p-value
Walking	20.0	62.5	17.5	0.730 (0.652, 0.807)	0.856	<0.0001
Arms	22.5	61.7	15.8	0.479 (0.341, 0.617)	0.552	<0.0001
Vision	23.3	56.7	20.0	0.342 (0.198, 0.486)	0.426	<0.0001
Speech	10.0	79.2	10.8	0.307 (0.115, 0.500)	0.367	<0.0001
Swallowing	8.3	85.0	6.7	0.419 (0.210, 0.627)	0.506	<0.0001
Cognitive	27.1	45.8	27.1	0.338 (0.205, 0.471)	0.446	<0.0001
Sensory	37.0	41.2	21.8	0.320 (0.196, 0.443)	0.414	<0.0001
Composite Score	47.5	16.9	35.6	0.462 (0.363, 0.554)	0.693	<0.0001

When we compared physician-derived MSRS scores with disease severity measures routinely captured at the Partners MS Center, there was good correlation between the composite MSRS and EDSS (r_s_ = 0.838, N = 117,), Timed 25 foot walk (T25FW) (r_s_ = 0.703, N = 98), and ambulation index (AI) (r_s_ = 0.813, N = 107). In terms of subdomains, the MSRS walking domain demonstrated a similar level of correlation with EDSS (r_s_ = 0.807), T25FW (r_s_ = 0.693), and AI (r_s_ = 0.840) (p<0.001 for all). We also compared patient-derived MSRS composite scores with physician-derived clinical scales; while the correlations were weaker, these remained highly significant (p<0.001): EDSS (r_s_ = 0.609), T25FW (r_s_ = 0.497), and AI (r_s_ = 0.591). Correlations with the MSRS walking domain score were somewhat stronger: EDSS (r_s_ = 0.738), T25FW (r_s_ = 0.650), and AI (r_s_ = 0.738).

### III. Investigation of BMI and Disease Course

Having established the clinical relevance of the MSRS, we analyzed prospectively collected data from PLM members to assess the role of BMI in disease severity, as assessed by self-reported MSRS. At last data entry, the mean BMI among PLM members was 27.6 kg/m^2^ (n = 10,433), with 25.7% subjects who were overweight (BMI>24.9) and 26.7% subjects who were obese (BMI>29.9) (**Table 3**).

**Table 3:** Distribution of self-reported BMI in the PLM population.

BMI Distribution	Within 3 monthsof first symptom	Last data entry
N	1173	10433
Underweight	42 (3.6%)	330 (3.2%)
Normal	476 (40.6%)	4058 (38.9%)
Overweight	313 (26.7%)	2970 (28.5%)
Obese	342 (29.2%)	3075 (29.5%)
Mean (SD)	27.36 (6.78)	27.56 (6.78)
Median	25.84	26.2

Cross-sectionally, we noted a very modest correlation (rho = 0.17, n = 1336) between greater BMI (continuous variable) and greater MSRS (**Table 4**). While significant because of the large sample size (p<0.001), the magnitude of this correlation is sufficiently small that it is unlikely to be of clinical relevance. Using a mixed model approach, we also assessed whether, in patients who have MS, BMI at a given time affects the subsequent trajectory of disease severity as assessed by repeated MSRS measures. In this analysis of prospectively collected MSRS, we did not see an effect of BMI on MSRS trajectory (estimate (95% CI) = 0.0133 (−0.00013, 0.0267), p = 0.05, n = 1695), after adjusting for disease duration, age, sex, race, disease type, and the interaction between disease type and follow-up time. When available (n = 236), we also assessed the relation of reported BMI at symptom onset with subsequent MSRS trajectory, and we also found no effect of BMI at symptom onset with subsequent disease course (estimate (95% CI) = 0.0137 (−0.0162, 0.0436), p = 0.37, n = 236). Overall, these longitudinal analyses – along with the cross-sectional analysis - suggest that BMI does not have a strong effect on MS disease severity as estimated by the self-reported MSRS instrument.

**Table 4:** Cross-sectional and longitudinal associations of BMI with disease severity, as measured by the MSRS.

	statistic	p-value
**Cross-sectional association**		
Adjusted Spearman correlation between BMI at last data entry and MSRS (N = 6229)*	Adjusted r = 0.14	<0.001
**Longitudinal association**		
Interaction term between BMI within 3 months of symptom onset and follow-up time (N = 236)**	Estimate = 0.0137 95% CI = −0.0162, 0.0436	0.37
Interaction term between BMI at first data entry and follow-up time (N = 1695)**	Estimate = 0.0133 95% CI = −0.00013, 0.0267	0.05

* All models adjusted for current age, sex, disease duration (years), disease course, and race.

** Models also adjusted for interaction between disease type and follow-up time.

## Discussion

In this study, we report good correlation of the patient-reported MSRS measure developed by PLM with standard clinical assessment measures used in MS, such as the EDSS. The availability of this online instrument and of the large PLM membership provides a useful, complementary platform for MS clinical research. It does not replace rigorous, prospective cohort studies or targeted clinical research projects performed in academic MS centers; instead, it offers a different option, that has an opportunity to leverage the frequent sampling of data from a large subject population.

While we found small demographic differences between PLM members with recent profile updates and patients at our MS center, they were modest in absolute terms. It should be noted that both sets of subjects represent somewhat biased subsets of the overall MS patient population; this limitation is somewhat mitigated by their large sample size but needs to be clearly stated. Interestingly, the differences that we describe between the patient populations we compared – including that PLM members had a younger age, higher education, larger proportion of women and lower proportion of self-reported white non-Hispanic origin - are slightly at odds with other demographic surveys of users of online tools, that have noted greater use and more frequent use by individuals who are white, female, older, with higher incomes, with good internet skills, and who are not employed [20]. We limited our comparative analysis to the subset of PLM members with recent profile updates, in order to examine active members who are likely to participate in ongoing online investigations; however, it is possible that these individuals differ in demographic terms from the general PLM population. The greater proportion of interferon beta-treated PLM members may partially reflect the fact that they had more relapsing onset disease. We also compared the PLM population to the 31,232 respondents from NARCOMS, a large and well-described registry-based cohort [21], [22] (**Table S5**). Here, we found statistically significant but small absolute differences in demographic characteristics, further suggesting that the PLM members, offer a reasonable alternative platform for MS clinical research that could yield insights that are generalizable to other MS populations after appropriate control for confounders.

In this study, we explored the use of PLM to answer a timely clinical question in which the current literature offers conflicting results: the relationship between excess body weight and MS disease course in the North American population that is becoming increasingly heavier. Obesity in an individual’s mother [23] or during adolescence of the individual [5], [6] may increase the risk for MS and perhaps for a relapsing onset [7]. However, in adulthood, insofar as it is possible to disentangle the effects of age, disability and comorbidities, individuals with MS may have lower body mass index (BMI) than age-matched controls [8], [24]–[27]. Only one prior study, by the North American Research Committee on Multiple Sclerosis (NARCOMS), examined longitudinal data and suggested that fluctuations in BMI are not associated with changes in disease severity as measured by Patient-determined disease steps [8]. Here, using a large sample size and prospectively collected data, we report consistent results suggesting that there is no evidence for increased BMI having a strong effect on disability in MS: we noted a very modest correlation between BMI and MSRS in the cross-sectional analysis (rho = 0.17, n = 1336) and only a nominally significant association in the analysis of the longitudinal MSRS data (estimate (95% CI) = 0.0133 (−0.00013, 0.0267)), suggesting that there is no clinically meaningful role for BMI in disability for MS in most individuals. Individuals do tend to under-estimate their BMI in self-reports [28]; however, because we examined BMI as a continuous variable this under-estimation should not influence the statistical estimate of this relationship on longitudinal course. However, we cannot exclude the possibility that BMI may have a very weak effect on the accumulation of disability, that it may influence other measures of disability in MS or that it may have a role in a subset of individuals. Further, the adverse health consequences of high BMI are well documented and doubtlessly impact the life course of MS patients [7], irrespective of a direct effect of BMI on MS disability.

Our findings and those of others relating BMI and disease severity in adults [8], contrast with the increased risk of MS associated with higher BMI during adolescence noted in prior studies [5], [6], possibly through immunologic modulation by hormones such as leptin and adiponectin [29]–[32]. This disjunction of a risk factor’s role in susceptibility and disease course has been suggested for other susceptibility factors such as EBV infection [33] and genetic risk factors [34], but not for cigarette smoking [35], [36] and vitamin D levels [37], which may influence both disease susceptibility and disease course. Additionally, because high BMI is associated with low vitamin D status, the association between BMI and risk of MS may in fact be confounded by vitamin D status [38]. As we better understand the functional consequences of these various risk factors, we may begin to see how they influence downstream molecular events leading to MS onset and/or disability.

There are challenges and limitations to online research platforms, including biases arising from the digital divide, issues of privacy, autonomy and data storage, variable and inconsistent data sampling schedules, and lack of objective validation of reported data such as height and weight [39], [40]. Also, in the absence of a gold standard for patient reported outcomes, it is not feasible to determine whether the physician or patient is “right”, only to understand the broader areas of agreement and discordance. For example, a patient may be more aware of sensory disturbances, but a clinician may better appreciate the broader range of gait disturbance seen in clinical practice. In this study a bias towards greater MSRS agreement between provider and patient may have existed, as the provider relied on the patient during the clinical interview to answer some questions, such as those pertaining to swallowing. Also, as with any clinical instrument, test-retest reliability is critical to enable powerful analysis; while we did not assess this feature of the MSRS as part of our study, the reliability of the MSRS has been previously evaluated and revealed one-week test-retest correlations of r = 0.91 (p<0.001) [1]. While not as comprehensive as a clinical examination or as biomedically grounded as an MRI scan, a patient reported outcome of disability such as the MSRS has the advantage of being rapid and free to administer; it also has the potential to enable patients themselves to learn more about the progression of their illness through self-monitoring. Further, missing data and incomplete participation by many members are important limitations that require rigorous quality control measures such as the ones that we implemented. In this study, from an initial 28,025 members reporting at least some information pertaining to MS, limiting the analysis to subjects with at least two separate dates of data entry in the study period and implementing the other quality measures reduced the analysis to a final N of 10,255 subjects. Finally, because online platforms are relatively new, the follow up time may not yet be sufficient long to detect clinically meaningful changes in the MSRS.

In conclusion, online research remains in its infancy and continues to pose ethical and methodological challenges. Nonetheless, the opportunity of capturing an assessment of disability on a very frequent basis opens exciting new possibilities that are not possible using more episodic data collection strategies such as those collected by NARCOMS. Prospective tracking of symptoms in “real time” may eventually allow researchers to assess whether certain symptoms such as increases in fatigue or fluctuations in “brain fog” can serve as harbingers for clinical attacks or be useful in assessing a patient’s clinical trajectory or response to disease-modifying therapy. The PLM member population therefore offers an intriguing new platform for MS investigations. Its results, after appropriate consideration of potential demographic and other confounders, may be generalizable to and replicable in the larger patient population seen in large MS centers.

## Supporting Information

Figure S1 **Bland-Altman plot for Patient and Physician Total MSRS scores.**
(DOCX)

Table S1 **Illustration: The Multiple Sclerosis Related Severity (MSRS) questionnaire.**
(DOCX)

Table S2 **Comparison of patient and disease characteristics for patients treated with glatiramer acetate and interferons.**
(DOCX)

Table S3 **Individual and Disease Characteristics of 121 MS Center patients completing MSRS questionnaire.**
(DOCX)

Tables S4a–g **Distribution of patient and physician scores for individual patients.**
(DOCX)

Table S5 **Comparison of PLM members' individual and disease characteristics with those of patients followed in the NARCOMS Registry.**
(DOCX)
