# Supplementary figures and images for: Evaluation of an Online Platform for Multiple Sclerosis Research: Patient Description, Validation of Severity Scale, and Exploration of BMI Effects on Disease Course
Source: PLoS One. 2013 Mar 20;8(3):e59707. doi: 10.1371/journal.pone.0059707 (PMC3603866; doi:10.1371/journal.pone.0059707)

**Figure S1. Bland-Altman plot for Patient and Physician Total MSRS scores**


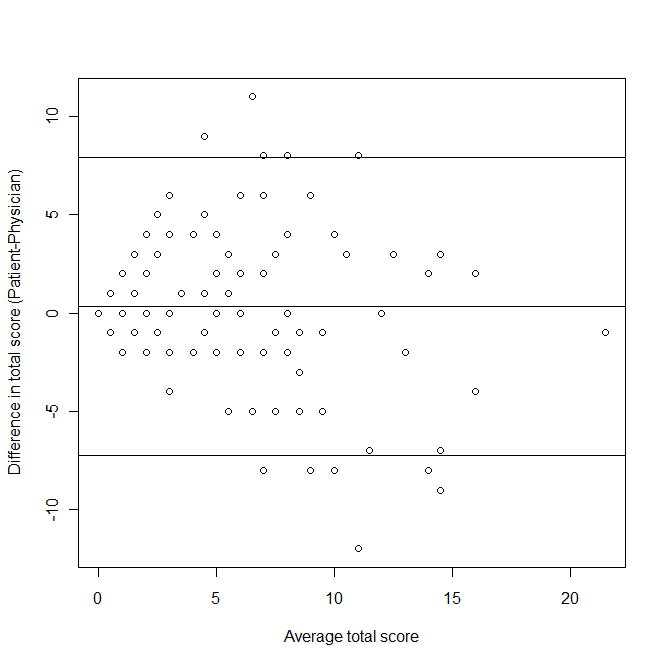

Supplement: Figure S1 — Bland-Altman plot for Patient and Physician Total MSRS scores. (DOCX) [file pone.0059707.s001.docx]
