# Supplementary material for: Evaluation of an Online Platform for Multiple Sclerosis Research: Patient Description, Validation of Severity Scale, and Exploration of BMI Effects on Disease Course
Source: PLoS One. 2013 Mar 20;8(3):e59707. doi: 10.1371/journal.pone.0059707 (PMC3603866; doi:10.1371/journal.pone.0059707)
Supplement: Table S1 — Illustration: The Multiple Sclerosis Related Severity (MSRS) questionnaire. (DOCX) [file pone.0059707.s002.docx]

**Table S1. Illustration: The Multiple Sclerosis Related Severity (MSRS) questionnaire.**

|  | **SCORE** | **0** | **1** | **2** | **3** | **4** |
| --- | --- | --- | --- | --- | --- | --- |
|  | **TITLE** | No Symptoms | Some symptoms, no disability | Mild disability | Moderate disability | Severe disability |
| **DOMAIN** | **TEXT** | "I have no symptoms or disability in this specific area" | "I am aware of symptoms but no limits on my activities" | "I have mild limits on my activities, but I do not need help from others or to use other aids" | "I have moderate limits on my activities and I sometimes need help from others or use other aids" | "I have severe limits on my activities and I usually need help from others or use other aids" |
| Walking |  |  |  |  |  |  |
| Using your arms and hands |  |  |  |  |  |  |
| Vision |  |  |  |  |  |  |
| Speech |  |  |  |  |  |  |
| Swallowing |  |  |  |  |  |  |
| Thinking, memory or cognition |  |  |  |  |  |  |
| Numbness, tingling, burning sensation or pain |  |  |  |  |  |  |

For each of the 7 domains, a subject will select one of the cells in the row for that domain, ranging from a score of “0” (no symptoms) to “4” (severe disability).
