# Supplementary material for: Evaluation of an Online Platform for Multiple Sclerosis Research: Patient Description, Validation of Severity Scale, and Exploration of BMI Effects on Disease Course
Source: PLoS One. 2013 Mar 20;8(3):e59707. doi: 10.1371/journal.pone.0059707 (PMC3603866; doi:10.1371/journal.pone.0059707)
Supplement: Table S2 — Comparison of patient and disease characteristics for patients treated with glatiramer acetate and interferons. (DOCX) [file pone.0059707.s003.docx]

**Table S2. Comparison of patient and disease characteristics for patients treated with glatiramer acetate and interferons**

|  | | **PLM** | | **MS Center** | **COMPARISON** | **METHOD** |
| --- | --- | --- | --- | --- | --- | --- |
| **GLATIRAMER ACETATE** | | | | | | |
| **N** | **2552** | | **987** | | **p-value** |  |
| **Current Age** | 44.65 (10.16) | | 47.21 (11.26) | | < 0.001 | t-test |
| **Age at first symptom** | 33.29 (9.84) | | 34.85 (10.24) | | < 0.001 | t-test |
| **Disease Duration, yrs** | 11.34 (8.91) | | 12.36 (9.67) | | 0.0043 | t-test |
| **Gender (F)** | 2069 (82.10) | | 707 (72.29) | | < 0.001 | chi-sq |
| **Family History** | 527 (21.22) | | 231 (24.65) | | 0.0313 | chi-sq |
| **Race** |  | |  | | 0.2007 | Fisher's exact |
| Native American | 7 (1.00) | | 2 (0.21) | |  |  |
| Asian | 4 (0.57) | | 7 (0.74) | |  |  |
| Black | 25 (3.58) | | 29 (3.07) | |  |  |
| Mixed Race | 18 (2.58) | | 18 (1.91) | |  |  |
| Hawaiian | 0 (0) | | 0 (0) | |  |  |
| White | 645 (92.27) | | 888 (94.07) | |  |  |
| **MS course** |  | |  | | < 0.001 | Fisher's exact |
| Primary progressive | 106 (4.44) | | 36 (4.05) | |  |  |
| Secondary progressive | 217 (9.08) | | 148 (16.67) | |  |  |
| Progressive relapsing | 57 (2.39) | | 3 (0.34) | |  |  |
| Relapsing-remitting | 2009 (84.09) | | 701 (78.94) | |  |  |
| **Education** |  | |  | | < 0.001 | Fisher's exact |
| 8th Grade or Less | 2 (0.28) | | 7 (0.84) | |  |  |
| Some High School | 13 (1.85) | | 16 (1.92) | |  |  |
| High School Grad | 92 (13.11) | | 118 (14.13) | |  |  |
| Some College | 299 (42.59) | | 465 (55.69) | |  |  |
| College | 185 (26.35) | | 112 (13.41) | |  |  |
| Post Graduate | 111 (15.81) | | 117 (14.01) | |  |  |
| **INTERFERONS** | | | | | | |
| **N** | **2998** | | **737** | | **p-value** |  |
| **Current Age** | 43.92 (10.42) | | 46.55 (10.96) | | < 0.001 | t-test |
| **Age at first symptom** | 32.86 (9.83) | | 33.68 (9.89) | | 0.0458 | t-test |
| **Disease Duration, yrs** | 11.09 (8.77) | | 12.72 (8.94) | | < 0.001 | t-test |
| **Gender (F)** | 2374 (79.66) | | 530 (72.50) | | < 0.001 | chi-sq |
| **Family History** | 638 (21.91) | | 158 (22.48) | | 0.7453 | chi-sq |
| **Race** |  | |  | | 0.3792 | Fisher's exact |
| Native American | 3 (0.43) | | 1 (0.15) | |  |  |
| Asian | 4 (0.58) | | 8 (1.16) | |  |  |
| Black | 46 (6.64) | | 42 (6.11) | |  |  |
| Mixed Race | 21 (3.03) | | 13 (1.89) | |  |  |
| Hawaiian | 1 (0.14) | | 0 (0) | |  |  |
| White | 618 (89.18) | | 623 (90.68) | |  |  |
| **MS course** |  | |  | | <0.001 | chi-sq |
| Primary progressive | 113 (4.10) | | 13 (1.90) | |  |  |
| Secondary progressive | 254 (9.22) | | 89 (12.99) | |  |  |
| Progressive relapsing | 68 (2.47) | | 8 (1.17) | |  |  |
| Relapsing-remitting | 2320 (84.21) | | 575 (83.94) | |  |  |
| **Education** |  | |  | | < 0.001 | Fisher's exact |
| 8th Grade or Less | 1 (0.14) | | 3 (0.48) | |  |  |
| Some High School | 10 (1.42) | | 8 (1.27) | |  |  |
| High School Grad | 100 (14.20) | | 115 (18.25) | |  |  |
| Some College | 302 (42.90) | | 349 (55.40) | |  |  |
| College | 179 (25.43) | | 72 (11.43) | |  |  |
| Post Graduate | 112 (15.91) | | 83 (13.17) | |  |  |
