# Supplementary material for: Evaluation of an Online Platform for Multiple Sclerosis Research: Patient Description, Validation of Severity Scale, and Exploration of BMI Effects on Disease Course
Source: PLoS One. 2013 Mar 20;8(3):e59707. doi: 10.1371/journal.pone.0059707 (PMC3603866; doi:10.1371/journal.pone.0059707)
Supplement: Table S3 — Individual and Disease Characteristics of 121 MS Center patients completing MSRS questionnaire. (DOCX) [file pone.0059707.s004.docx]

**Table S3. Individual and Disease Characteristics of 121 MS Center patients completing MSRS questionnaire**

| **Characteristic** | **Result** |
| --- | --- |
| Age (yrs): mean (SD) | 47.1 (12.0) |
| Age first symptoms, yrs: mean (SD) | 33.7 (10.2) |
| Disease Duration, yrs: mean (SD) | 13.4 (10.6) |
| Gender (%) |  |
| Female | 73% |
| Male | 27% |
| Disease Category (%) |  |
| Relapsing-remitting | 68% |
| Secondary Progressive | 31% |
| Primary Progressive | 2% |
| EDSS Score (N=118) |  |
| Distribution (N) |  |
| 0 | 36 |
| 1-1.5 | 15 |
| 2-2.5 | 19 |
| 3-3.5 | 13 |
| 4-4.5 | 1 |
| 5-5.5 | 6 |
| 6-6.5 | 17 |
| 7-7.5 | 7 |
| 8-8.5 | 4 |
