# Supplementary material for: Evaluation of an Online Platform for Multiple Sclerosis Research: Patient Description, Validation of Severity Scale, and Exploration of BMI Effects on Disease Course
Source: PLoS One. 2013 Mar 20;8(3):e59707. doi: 10.1371/journal.pone.0059707 (PMC3603866; doi:10.1371/journal.pone.0059707)
Supplement: Tables S4a–g — Distribution of patient and physician scores for individual patients. (DOCX) [file pone.0059707.s005.docx]

**Tables S4a-g. Distribution of patient and physician scores for individual patients.**

a. Walking domain:

|  | **Physician score** | | | | |
| --- | --- | --- | --- | --- | --- |
| **Patient score** | **0** | **1** | **2** | **3** | **4** |
| **0** | 40 | 1 | 0 | 0 | 0 |
| **1** | 5 | 6 | 9 | 1 | 0 |
| **2** | 3 | 8 | 9 | 5 | 0 |
| **3** | 1 | 0 | 0 | 6 | 5 |
| **4** | 1 | 0 | 0 | 6 | 14 |

b. Upper extremity domain:

|  | **Physician score** | | | | |
| --- | --- | --- | --- | --- | --- |
| **Patient score** | **0** | **1** | **2** | **3** | **4** |
| **0** | 57 | 6 | 3 | 2 | 0 |
| **1** | 13 | 4 | 4 | 0 | 0 |
| **2** | 5 | 3 | 8 | 3 | 1 |
| **3** | 2 | 3 | 1 | 3 | 0 |
| **4** | 0 | 0 | 0 | 0 | 2 |

c. Vision domain:

|  | **Physician score** | | | | |
| --- | --- | --- | --- | --- | --- |
| **Patient score** | **0** | **1** | **2** | **3** | **4** |
| **0** | 58 | 6 | 5 | 1 | 0 |
| **1** | 17 | 6 | 4 | 3 | 1 |
| **2** | 7 | 1 | 3 | 2 | 0 |
| **3** | 0 | 2 | 1 | 0 | 2 |
| **4** | 0 | 0 | 0 | 0 | 1 |

d. Speech domain:

|  | **Physician score** | | | | |
| --- | --- | --- | --- | --- | --- |
| **Patient score** | **0** | **1** | **2** | **3** | **4** |
| **0** | 92 | 5 | 3 | 0 | 0 |
| **1** | 8 | 2 | 2 | 1 | 0 |
| **2** | 2 | 0 | 1 | 2 | 0 |
| **3** | 2 | 0 | 0 | 0 | 0 |
| **4** | 0 | 0 | 0 | 0 | 0 |

e. Swallow domain:

|  | **Physician score** | | | | |
| --- | --- | --- | --- | --- | --- |
| **Patient score** | **0** | **1** | **2** | **3** | **4** |
| **0** | 97 | 2 | 3 | 0 | 0 |
| **1** | 6 | 2 | 2 | 1 | 0 |
| **2** | 2 | 1 | 3 | 0 | 0 |
| **3** | 1 | 0 | 0 | 0 | 0 |
| **4** | 0 | 0 | 0 | 0 | 0 |

f. Cognitive domain:

|  | **Physician score** | | | | |
| --- | --- | --- | --- | --- | --- |
| **Patient score** | **0** | **1** | **2** | **3** | **4** |
| **0** | 38 | 14 | 5 | 1 | 0 |
| **1** | 15 | 10 | 8 | 1 | 0 |
| **2** | 1 | 5 | 4 | 1 | 0 |
| **3** | 1 | 6 | 2 | 1 | 2 |
| **4** | 1 | 0 | 0 | 1 | 1 |

g. Sensory domain:

|  | **Physician score** | | | | |
| --- | --- | --- | --- | --- | --- |
| **Patient score** | **0** | **1** | **2** | **3** | **4** |
| **0** | 22 | 11 | 2 | 3 | 0 |
| **1** | 16 | 13 | 5 | 1 | 0 |
| **2** | 5 | 9 | 10 | 3 | 0 |
| **3** | 4 | 3 | 3 | 4 | 1 |
| **4** | 0 | 0 | 2 | 2 | 0 |
