# Supplementary material for: Evaluation of an Online Platform for Multiple Sclerosis Research: Patient Description, Validation of Severity Scale, and Exploration of BMI Effects on Disease Course
Source: PLoS One. 2013 Mar 20;8(3):e59707. doi: 10.1371/journal.pone.0059707 (PMC3603866; doi:10.1371/journal.pone.0059707)
Supplement: Table S5 — Comparison of PLM members' individual and disease characteristics with those of patients followed in the NARCOMS Registry. (DOCX) [file pone.0059707.s006.docx]

**Table S5. Comparison of PLM members' individual and disease characteristics with those of patients followed in the NARCOMS Registry**

| **Variable** | **PLM** | **NARCOMS*** | **COMPARISON** | |
| --- | --- | --- | --- | --- |
|  | **N = 10255** | **N = 31232** | **p-value** | **Method** |
| Current Age, yrs: mean (SD) | 44.79 (10.56) | 47.04 (10.84) | < 0.001 | t-test |
| Age at first symptom, yrs: mean (SD) | 32.83 (10.01) | 29.22 (10.96) | < 0.001 | t-test |
| Gender (% F) | 80.1 | 72.5 | <0.001 | chi-sq |
| Race (%) |  |  | 0.0055 | Fisher's exact |
| Caucasian | 90.4 | 90.4 |  |  |
| African American | 5.1 | 4.4 |  |  |
| Other | 4.4 | 5.2 |  |  |
| Education (%) |  |  | - | - |
| Education Level, based on yrs |  |  |  |  |
| Less than 12 years | 2.2 |  |  |  |
| Completed High School | 14.7 |  |  |  |
| Some College | 42.2 |  |  |  |
| Completed College | 25.8 |  |  |  |
| Post Graduate | 15.1 |  |  |  |
| Highest degree achieved |  |  |  |  |
| Less than high school |  | 3.1 |  |  |
| High School Degree |  | 40.6 |  |  |
| Associate's or Technical Degree |  | 17.7 |  |  |
| Bachelor's Degree |  | 23.1 |  |  |
| Post Graduate |  | 15.7 |  |  |

NB. For individual variables, as some subjects had missing data, the N used for calculation of percentages was lower than the total N of respondents

*Published data from the NARCOMS Registry were taken from:

Buchanan RJ, Chakravorty BJ, Tyry T, Hatcher W, Vollmer T (2009) Age-related comparisons of people with multiple sclerosis: demographic, disease, and treatment characteristics. NeuroRehabilitation 25: 271-278.
